# The Enigma of Sphingolipids in Health and Disease

**DOI:** 10.3390/ijms19103126

**Published:** 2018-10-12

**Authors:** Burkhard Kleuser

**Affiliations:** Department of Toxicology, Institute of Nutritional Science, Faculty of Mathematics and Natural Science, University of Potsdam, Arthur-Scheunert Allee 114-116, 14558 Nuthetal, Germany; kleuser@uni-potsdam.de; Tel.: +49-33200-885301; Fax: +49-33200-885541

Sphingolipids are one of the major classes of eukaryotic lipids. Johann Ludwig Thudichum discovered them in 1874 by fractional crystallization of ethanolic brain extracts. The root term “sphingo-” was introduced by Thudichum according to the Greek mythical creature, the Sphinx, as the enigmatic nature of the molecules reminded him of the many enigmas the Sphinx presented to the inquirer [1]. Although this lipid class shows great structural diversity and complexity, the characteristic feature of all sphingolipids is the presence of a sphingoid backbone. In mammalian cells, this is normally sphingosine, (2*S*,3*R*,4*E*)-2-amino-4-octadecene-1,3-diol. For almost a century, sphingolipids were regarded only as structural components of lipid bilayers, such as biological membranes. However, investigations in the mid-1980s revealed that specific sphingolipid species are involved in the regulation of biological processes. The crucial bioactive sphingolipids that have gained the most interest are ceramides, ceramide 1-phosphate, glucosylceramide, sphingosine, and sphingosine 1-phosphate (S1P). A further milestone was passed with the discovery of five high-affinity G-protein coupled receptors (GPCR) for the sphingolipid metabolite S1P, explaining the variety of different actions of this sphingolipid through canonical GPCR signaling [2].

In the special issue *Sphingolipids: Signals and Disease*, promising findings of this enigmatic lipid class are depicted, covering many functions of sphingolipids in physiological and pathophysiological conditions. A growing accretion of the role of sphingolipids in health and disease is driven by the translation from basic cell studies to animal experiments and human clinical studies; all aspects are covered in this special issue.

Defects in the metabolism of sphingolipids were discovered as lysosomal storage disorders in humans. Moskot et al. presented an overview of our current understanding of dysregulation of sphingolipid metabolism in diseased mammalian systems [3].

A fundamental role of sphingolipids in the function of the immune system has been established. S1P is pivotally important in immune cell trafficking [4]. T cell emergence is controlled by a gradient of S1P between the lymph and the plasma via the S1P receptor subtype 1 (S1PR1). Repeated administration of S1PR1 modulators triggers a sustained internalization of this receptor, which is accompanied by a long-lasting inhibition of the emergence of lymphocytes from lymphoid organs. This immunosuppressive effect is considered a therapeutic approach to treat autoimmune diseases and has been directly applied in the treatment of multiple sclerosis with the non-selective S1P receptor modulator fingolimod (Gilenya^®^) [5]. In the special issue and for the first time, Juif et al. presented the pharmacokinetics, pharmacodynamics, safety, and tolerability of cenerimod, a novel selective S1PR1 modulator, in healthy humans [6]. In addition to multiple sclerosis, T cell activation plays a pivotal role in Graft-versus-Host disease (GvHD) following allogenic haemopoietic stem cell transplantation. It can be speculated that pharmacological intervention via S1P modulation may have the potential to improve patient outcome by regulating GvHD and enhancing the engraftment [7]. S1P also regulates the migration of follicular B cells and directs the positioning of Marginal zone B cells within the spleen. The role of S1P in the third B cell lineage, mainly present in the peritoneal cavity, has not been well investigated. Kleinwort et al. indicated that S1P signaling affects peritoneal B cell migration. Most interestingly, this effect is mediated via the S1PR4 subtype. As peritoneal B cells are the major source of natural serum immunoglobulin (Ig) A, downregulation of S1PR4 reduces the production of intestinal IgA [8]. A critical role of S1PR4 has been detected in mast cells. Olivera et al. were able to show that, although the S1PR4 is dispensable for mast cell degranulation and cytokine production, this receptor subtype regulates passive systemic anaphylaxis in mice [9].

Bioactive sphingolipids have emerged as key players in cancer cell biology even in a divergent manner. Ceramides have been found to inhibit cancer cell proliferation and to promote cancer cell apoptosis; S1P has been attributed to cell growth and cell survival. Growing evidence has demonstrated that sphingosine kinase (SphK) and S1P signaling are dysregulated in cancers, such as colorectal cancer [10] and glioblastoma multiforme [11]. The relevance of aberrant dicing and splicing of SphK isozymes and the production of variant SphK isoforms in the development and progression of malignancy were discussed by Haddadi et al. [12]. SphK/S1P signaling could be a useful target in the therapy of several cancer types. 1,2,3-triazole-based SphK inhibitors were developed by Severino et al. [13].

Convincing data indicate a role for sphingolipids in modulating metabolic functions, and changes in levels of specific sphingolipid species have been implicated in metabolic disorders. Thus, the role of S1P in liver diseases, such as hepatic insulin resistance or fibrosis, was reviewed [14]. However, it is of interest that apolipoprotein M (apoM) acts as a chaperone to transport S1P. The role of apoM in liver fibrosis was illuminated [15]. Kidney-derived apoM seems to play an essential role in S1P recovery to prevent urinal loss. The kidney is an organ sensitive to sphingolipid alterations, which may contribute to numerous nephropathic complications [16]. S1P was discussed as a crucial molecule to initiate renal fibrosis. The S1P transporter spinster homology protein 2 seems to be involved in this pathophysiological condition [17].

As alterations in sphingolipid levels contribute to the initiation and progression of several diseases, it is not astonishing that sphingolipids may serve as biomarkers that identify dysfunction of physiological processes [18,19].

Moreover, it is well established that intensive crosstalk occurs between sphingolipids and other lipid classes. Bernacchioni et al. described a novel interaction between ceramide 1-phosphate and lysophosphatidic acid in skeletal muscle, implying a synergistic role of both lipids in muscle regeneration [20].

In addition to the above-mentioned topics, the special issue includes further distinguished reviews and articles that illuminate the role of sphingolipids in organs and tissues, such as the lung, the skin, the muscle, and the retina. Taken together, this special issue provides new insights into the role of sphingolipid metabolism in health and disease.

Finally, I would like to thank all of the authors for their impressive manuscripts and all of the referees for their remarkable efforts in supporting this special issue.
